# The association of bariatric surgery and Dupuytren’s disease: a propensity score-matched cohort study

**DOI:** 10.1177/17531934211062023

**Published:** 2021-12-01

**Authors:** Theresa Burkard, Jennifer C. E. Lane, Dag Holmberg, Anders Thorell, Andrea M Burden, Dominic Furniss

**Affiliations:** 1 Department of Chemistry and Applied Biosciences, ETH Zurich, Zurich, Switzerland; 2 Nuffield Department of Orthopaedics, Rheumatology and Musculoskeletal Sciences, University of Oxford, Oxford, UK; 3 Upper Gastrointestinal Surgery, Karolinska University Hospital, Stockholm, Sweden; 4 Department of Clinical Science, Danderyd Hospital, Stockholm, Sweden; 5 Department of Surgery, Ersta Hospital, Stockholm, Sweden

**Keywords:** Dupuytren’s disease, bariatric surgery, cohort study, body mass index, obesity

## Abstract

We aimed to assess the association between bariatric surgery and incident Dupuytren’s disease (DD) using propensity score-matched cohort studies among Swedish nationwide healthcare registries. Patients aged 30–79 years who underwent bariatric surgery 2006–2019 were matched on their propensity scores, up to two obese bariatric surgery-free (unexposed) patients. We applied Cox proportional hazard regression to calculate hazard ratios (HR) with 95% confidence intervals (CI) for the risk of DD overall, in subgroups of age, sex, bariatric surgery type and duration of follow-up. Among 34,959 bariatric surgery patients and 54,769 propensity score-matched obese patients, the risk of DD was increased in bariatric surgery patients compared with obese unexposed patients (HR 1.30, 95% CI 1.02–1.65), among women (HR 1.36; 1.00–1.84); those undergoing gastric bypass (HR 1.33; 1.04–1.71) and those with >5 years follow-up (HR 1.63; 1.14–2.34). Our results suggest that substantial weight loss is associated with an increased risk of DD in an obese population.

**Level of evidence:** III

## Introduction

The association between obesity and Dupuytren’s disease (DD) is highly biologically unusual, being one of only two diseases where obesity is found to be protective in the development of the disease (Majeed et al., 2021). Large epidemiological cohort studies have suggested that obesity is protective for DD (Gudmundsson et al., 2000; Hacquebord et al., 2017; Kuo et al., 2020). Furthermore, genome wide association and Mendelian randomization studies have also suggested a causally protective effect of increased body mass index (BMI) (Majeed et al., 2021; Major et al., 2019). However, other research has suggested an increased incidence of DD associated with diabetes mellitus and hyperlipidaemia, therefore complicating the relationship between DD, obesity and metabolic syndrome (Alser et al., 2020; Geoghegan et al., 2004; Hart and Hooper, 2005).

Weight loss is promoted in order to prevent sequelae of obesity, including diabetes mellitus, cardiovascular disease or cancer (WHO, 2020). Bariatric surgery is an effective treatment for obesity where lifestyle interventions alone have failed (Eaton et al., 2016). The majority of weight loss has been shown to occur within the first 2 years after surgery, with an established reduction in cardiovascular and inflammatory conditions, depression, cancer and mortality (Buchwald et al., 2004; Gill et al., 2019; Schauer et al., 2019).

While epidemiological and genetic studies of obesity and DD have been undertaken, the impact of weight loss following bariatric surgery among obese patients on incident DD has not been assessed to date. Modelling weight loss in epidemiological data is extremely challenging due to poor measurement, but bariatric surgery is frequently used as a surrogate as this is associated with immediate and sustained weight loss in patients. Therefore, we aimed to assess the impact of bariatric surgery on the incidence of DD in a large cohort of patients from national Swedish healthcare registries. Using propensity score-matching, we compared new-onset DD in obese patients who had undergone bariatric surgery with matched individuals who had not undergone bariatric surgery.

## Methods

### Data sources

We conducted a propensity score-matched sequential cohort study using data from the national Swedish healthcare registries, including the Patient Registry, Causes of Death Registry, Prescribed Drug Registry, Cancer Registry and the Scandinavian Obesity Surgery Registry (SOReg) (Brooke et al., 2017; Ludvigsson et al., 2011; SOReg, 2021). All individuals born or permanently residing in Sweden are assigned a 10-digit personal code that is used for identification in healthcare registries and allowed for linkage (Ludvigsson et al., 2009). The quality of the data on surgery has been externally validated within the Swedish patient registry, and excellent validity of bariatric surgery in the Swedish Patient Registry and SOReg has also been confirmed when compared against medical records (Ludvigsson et al., 2011; Tao et al., 2016). The Patient Registry was therefore used to identify bariatric surgery patients and SOReg to obtain details on the type of surgery codes and BMI measurements.

### Study population

**Figure 1.**
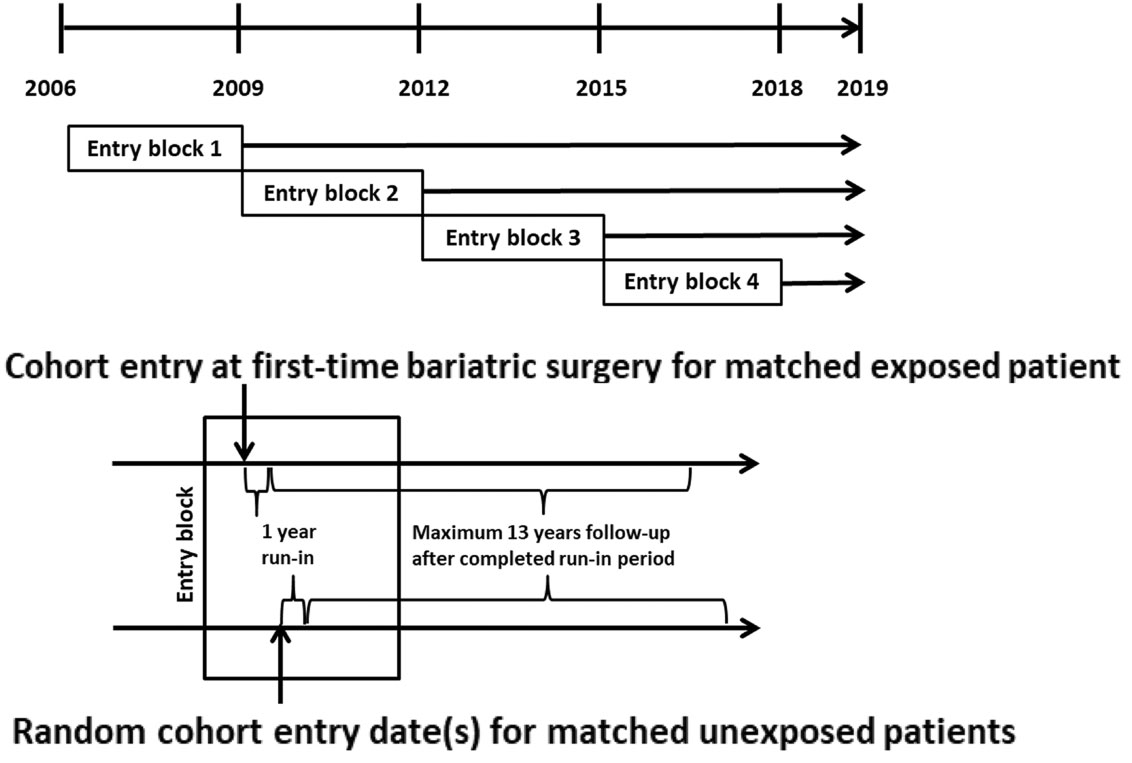
Study overview. (a) Each entry block represented one cohort. The cohorts contained all eligible exposed patients and their (1 to 2) PS-matched unexposed patients. (b) Detailed representation of each individual entry block. Matched exposed patients entered on the date of their surgery; matched unexposed patients entered on a random date within the entry block. We followed all patients for a maximum of 13 years after their completed run-in period of 1 year, until they had a record of DD or they were censored due to change in exposure status, death, loss to follow-up, occurrence of an exclusion criterion or end of study period.

We identified all individuals diagnosed with obesity (International Classification of Disease (ICD) version 10 code E66, ICD version 9 278 A/B, ICD version 8 287,0, ICD version 7 277,99) or bariatric surgery aged 30–79 years at any time between January 2006 and December 2019 in the Swedish Patient Registry (WHO, 2016). We excluded patients with a record of previous bariatric surgery in order to only include those undergoing primary weight-reducing surgery, DD diagnosis or surgery for DD prior to cohort entry (Figure 1).

### Study design

#### Exposures

Bariatric surgery was defined using NOMESCO (Nordic Medico-Statistical Committee) classification of surgical procedures codes (as of 1997: gastric bypass: JDF10–11; duodenal switch: JFD03–04; others: 93% sleeve gastrectomy according to SOReg, thus, we further referred to this group as sleeve gastrectomy: JDF00–01, JDF20–21, JDF96–97) (Nordic Co-operation, 2021). Patients were considered ‘exposed’ to bariatric surgery for their entire follow-up unless the surgical procedure was reversed to their original gastroenterological anatomy (NOMESCO code JFD23), in which case the follow-up ended. ‘Unexposed’ patients were defined as those identified with an ICD code for obesity but who had not undergone bariatric surgery. If an unexposed patient subsequently underwent bariatric surgery after entry into the cohort, they were censored at this stage and may have been eligible to enter the study as an exposed patient within the entry block design.

#### Outcome

We defined DD as the first recorded diagnosis of ICD-10 M72.0 or DD surgery (NOMESCO code of NDM09/19).

#### Entry block design

An illustration of the sequential block entry design is provided in Figure 1. We categorized exposed patients into one of four 3- to 4-year entry blocks according to the date of bariatric surgery, referred to as cohort entry (Figure 1(a)). Within each entry block, we additionally identified the eligible group of unexposed patients aged 30–79 years. These patients were assigned one or more random entry dates within the respective entry block (Figure 1(b)). Thus, patients could contribute one episode as an exposed patient but multiple episodes as an unexposed patient throughout the study period as their exposure to surgery changed.

### Follow-up

Follow-up began on day 365 after cohort entry (i.e. run-in period) as no earlier effect of weight loss following bariatric surgery was clinically expected. We followed all patients in an ‘as-treated’ approach until the first occurrence of DD or censoring due to occurrence of an exclusion criteria, change of bariatric surgery exposure status, loss to follow-up, death or end of study period (December 2019).

### Statistical methods

#### Propensity score

We estimated a propensity score (i.e. probability of undergoing bariatric surgery) for each exposed and unexposed patient using multivariable logistic regression. All covariates were selected a priori based on clinical knowledge (Hernán et al., 2002). Table 1 details all covariates used for matching (further information provided in Supplementary Appendix Methods S1). A greedy 8-1 digit matching algorithm without replacement was applied, excluding those who could not be matched (Parsons, 2001). We matched each exposed patient with up to two unexposed patients. In a sensitivity analysis, we trimmed our study population asymmetrically at the extreme ends of the propensity score tail (bariatric surgery patients below the 5th and unexposed patients above the 95th percentile before matching) to exclude exposed and unexposed patients treated contrary to prediction.

**Table 1.** Baseline characteristics of bariatric surgery patients and unexposed patients with obesity (follow-up >365 days) before and after PS-matching.

	Before PS-matching		PS-matched	
	Exposed (*N* = 43,870)	Unexposed (*N* = 317,205)	Exposed (*N* = 34,959)	Unexposed (*N* = 54,769)
Mean age [years] (SD)	44.6 (8.8)	54.3 (13.7)	45.5 (9)	45.9 (9.4)
Mean follow-up [years] (SD)	6.8 (3.1)	5.9 (3.3)	6.9 (3.2)	6.3 (3.3)
Female	32,581 (74.3%)	200,605 (63.2%)	25,036 (71.6%)	39,106 (71.4%)
Male	11,289 (25.7%)	116,600 (36.8%)	9923 (28.4%)	15,663 (28.6%)
Alcohol proxy	299 (0.7%)	8410 (2.7%)	296 (0.9%)	542 (1%)
Smoking or COPD	1276 (2.9%)	24,987 (7.9%)	1192 (3.4%)	1979 (3.6%)
Median number of hospital contacts ≤1 year before cohort entry date (IQR)	3 (2–4)	0 (0–3)	2 (2–4)	2 (0–5)
*Comorbidities before the cohort entry date:*				
Median Royal College of Surgeon Charlson comorbidity score (IQR)	0 (0–1)	1 (0–2)	0 (0–1)	0 (0–1)
Cholelithiasis/cholecystitis	1549 (3.5%)	18,415 (5.8%)	1440 (4.1%)	2361 (4.3%)
Deep vein thrombosis	169 (0.4%)	4038 (1.3%)	164 (0.5%)	315 (0.6%)
Diabetes Type 2^a^	7848 (17.9%)	96,596 (30.5%)	7195 (20.6%)	11,534 (21.1%)
Hyperlipidaemia^a^	5548 (12.7%)	94,100 (29.7%)	5175 (14.8%)	8506 (15.5%)
Hypertension^a^	11,214 (25.6%)	127,748 (40.3%)	9795 (28%)	15,366 (28.1%)
GERD^a^	21,394 (48.8%)	70,993 (22.4%)	14,119 (40.4%)	20,450 (37.3%)
Gout	254 (0.6%)	6567 (2.1%)	244 (0.7%)	412 (0.8%)
Hand trauma	787 (1.8%)	12,617 (4%)	757 (2.2%)	1322 (2.4%)
Ischemic heart disease^b^	630 (1.4%)	30,888 (9.7%)	618 (1.8%)	1165 (2.1%)
Menopause	983 (2.2%)	24,613 (7.8%)	958 (2.7%)	1773 (3.2%)
Migraine^a^	1537 (3.5%)	8270 (2.6%)	1330 (3.8%)	1998 (3.7%)
Pneumonia	548 (1.3%)	13,775 (4.3%)	526 (1.5%)	977 (1.8%)
Pregnancy/delivery	3548 (8.1%)	52,908 (16.7%)	3465 (9.9%)	5343 (9.8%)
Renal disease	376 (0.9%)	15,260 (4.8%)	357 (1%)	1186 (2.2%)
Sleep apnoea	5162 (11.8%)	48,661 (15.3%)	4535 (13%)	6976 (12.7%)
Stroke/transient ischemic attack	183 (0.4%)	11,532 (3.6%)	182 (0.5%)	374 (0.7%)
*Medications at the cohort entry date* *^c^* *:*				
Antibiotics	947 (2.2%)	6031 (1.9%)	739 (2.1%)	1029 (1.9%)
Antipsychotics	913 (2.1%)	11,333 (3.6%)	863 (2.5%)	1529 (2.8%)
Antidepressants	8649 (19.7%)	57,148 (18%)	7351 (21%)	11,300 (20.6%)
Anxiolytics	2858 (6.5%)	29,970 (9.5%)	2612 (7.5%)	4342 (7.9%)
Cardiovascular drugs	14,766 (33.7%)	173,983 (54.9%)	13,090 (37.4%)	20,590 (37.6%)
Hypnotics/sedatives	4736 (10.8%)	46,448 (14.6%)	4240 (12.1%)	6788 (12.4%)
*Cohort entry date* *^c^*				
2006–2008	4472 (10.2%)	43,693 (13.8%)	4161 (11.9%)	6731 (12.3%)
2009–2011	12,779 (29.1%)	66,388 (20.9%)	9694 (27.7%)	14,573 (26.6%)
2012–2014	13,549 (30.9%)	97,181 (30.6%)	11,103 (31.8%)	17,039 (31.1%)
2015–2018	13,070 (29.8%)	109,943 (34.7%)	10,001 (28.6%)	16,426 (30.0%)
Concordance-statistics	0.90		0.59	

PS: propensity score; COPD: chronic obstructive pulmonary disease; GERD: gastroesophageal reflux disease; IQR: interquartile range; NA: not available; SD: standard deviation.

a Diagnosis or medication.

b Includes myocardial infarctions and angina pectoris.

c Not used for propensity score matching.

#### Descriptive analysis

Stratified by exposure, we assessed patient characteristics at cohort entry before and after propensity score-matching. Furthermore, we assessed censoring reasons of the cohorts before and after matching and described cumulative incidences of DD stratified by exposure.

#### Regression analysis

After combining all sequential cohorts into one study cohort, we compared covariate distribution between treatment groups before and after propensity score-matching through estimation of standardized mean differences and the concordance-statistic (c-statistic). This c-statistic indicates the level of covariate balance between study groups where 0.5 indicates perfect balance and 1.0 indicates maximal imbalance (Franklin et al., 2014). Using Cox proportional hazard analyses, we estimated hazard ratios (HR) with 95% confidence intervals (CI) for the incidence of DD with bariatric surgery, compared with unexposed patients.

We performed subgroup analyses by sex, age (30–54 years, 55–79 years), bariatric surgery type (sleeve gastrectomy, gastric bypass, duodenal switch). For all subgroup analyses we re-matched within subgroups. The proportional hazard (PH) assumption was tested using the Martingale residual method, which did not hold in the overall analysis. Thus, we performed subgroup analyses by median follow-up (>1–5 years, >5–13 years). Since patients may contribute several episodes and matching may lead to correlated patient clusters, we estimated robust sandwich estimates for the covariate matrix (results remained unchanged).

As a sensitivity analysis as propensity score methodology is still considered novel by some, we also conducted all analyses using multivariable Cox regression in the unmatched cohort, adjusting for all covariates, included in the propensity scores.

## Results

### Demographics

**Figure 2.**
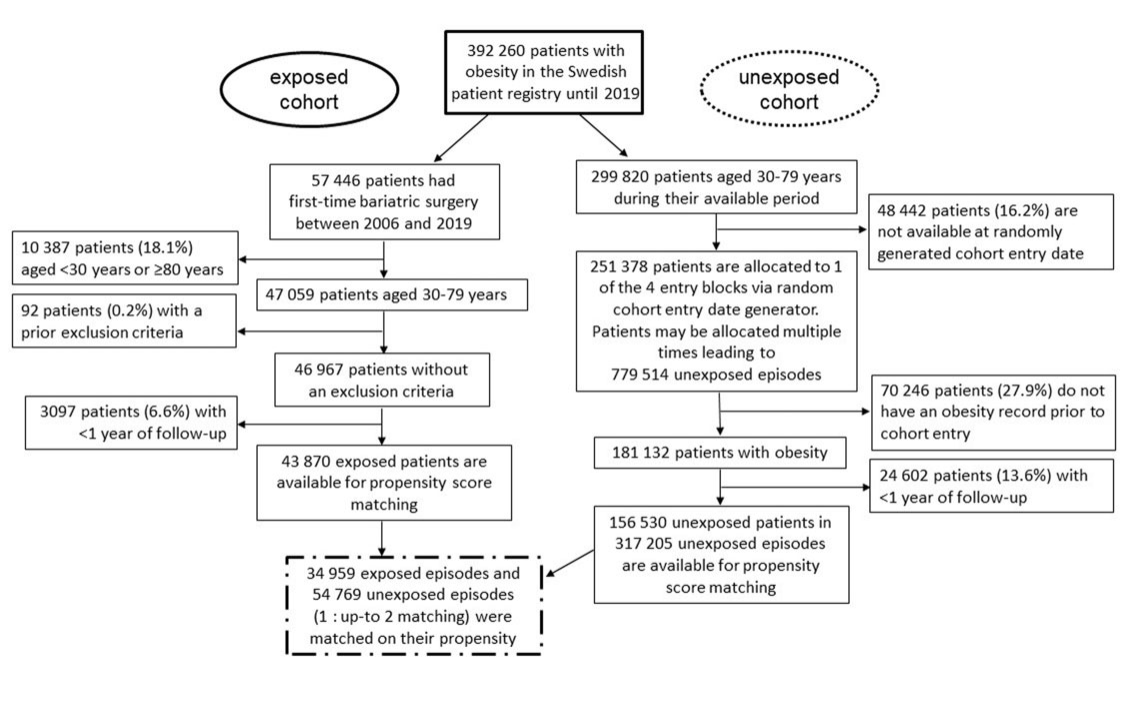
Flow-chart of the study composition.

Figure 2 shows the flowchart of data management. Of 43,870 exposed patients eligible for propensity score-matching, 34,959 (80%) were matched with up to two unexposed episodes resulting in a total of 89,728 matched episodes.

Before propensity score-matching, the average age of bariatric surgery patients was lower, they were more likely female, had more hospital contacts, were less frequently diagnosed with hypertension, type 2 diabetes or ischemic heart diseases, but more frequently diagnosed with gastroesophageal reflux disease (Table 1). Of those who underwent bariatric surgery, most patients underwent gastric bypass surgery (85%, Supplementary Table S1). Patients with duodenal switch had the highest mean BMI at surgery of 55.1 kg/m^2^. At 1, 2 and 5 years after surgery, BMIs were comparable between surgery types at around 30–33 kg/m^2^.

Supplementary Table S2 gives the details of follow-up and outcome. Before propensity score-matching, we observed 139 and 957 incident DD cases among exposed and unexposed patients, respectively. A total of 85% of DD cases were identified by a diagnosis code, 11% of cases were identified by a surgical code and 4% were identified by a combination of diagnosis and surgery (same day). In patients who had both a DD diagnosis and surgery code during the observation period, median time from diagnosis to surgery was 70 days (interquartile range 1–217 days).

After propensity score-matching, all patient characteristics of bariatric surgery patients and obese unexposed patients were highly similar (<10% of standardized mean differences between groups (Supplementary Figure S1), c-statistic of 0.59 (Table 1)). Moreover, censoring was comparable between groups after propensity score-matching.

### Risk of DD

Table 2 presents the association between bariatric surgery and DD overall, and stratified by our subgroup analyses. We observed an overall increased risk of DD in patients undergoing bariatric surgery compared with unexposed patients (HR 1.30, 95% CI 1.02–1.65). Moreover, we observed a higher risk of DD in women than in men, and in those who underwent gastric bypass than in those who underwent sleeve gastrectomy, when compared with unexposed patients. Furthermore, the risk of DD was similar among exposed and unexposed patients during the first 5 years of follow-up and increased thereafter. Extending the run-in period to 2 years and propensity scores trimming each accentuated the risk of incident DD. In order to evaluate the propensity score-matched approach, a Cox proportional hazard regression analysis adjusting for all covariates was also undertaken (Supplementary Table S3), which identified the same signal with regard to factors associated with increased risk of incident DD.

**Table 2.** Results of the association of bariatric surgery and DD overall and in subgroups, propensity score-matched analysis.

	Observation time^a^		DD events		
	Exposed	Unexposed	Exposed	Unexposed	HR matched^b^ (95% CI)
Overall	241.8	345.3	126	136	1.30 (1.02–1.65)
Sex					
Women	173.9	242.8	83	83	1.36 (1.00–1.84)
Men	66.1	99.5	40	56	1.05 (0.70–1.58)
Age in years					
30–54	194.4	267.8	79	77	1.38 (1.01–1.88)
55–79	42.5	8.6	42	52	1.37 (0.92–2.06)
Bariatric surgery type					
Sleeve gastrectomy	21.5	38.7	8	15	1.01 (0.43–2.38)
Gastric bypass	225.6	324.8	121	128	1.33 (1.04–1.71)
Duodenal switch	2.6	4.7	1	2	NA
Duration of follow-up					
>1–5 years	155.0	230.3	60	82	1.07 (0.76–1.49)
>5–13 years	76.8	101.4	66	54	1.63 (1.14–2.34)
Sensitivity analyses					
2-year run-in period	236	342.4	107	108	1.40 (1.07–1.83)
>2–5 years	149.9	225.5	44	54	1.20 (0.80–1.78)
Trimmed PS	127.5	220.1	69	78	1.49 (1.08–2.07)

DD: Dupuytren’s disease; PS: propensity score.

a Observation time in 1000 person-years (1000 people observed for 1 year would be 1000 person-years).

b Adjusted for PS estimation with covariates see Table 1.

**Figure 3.**
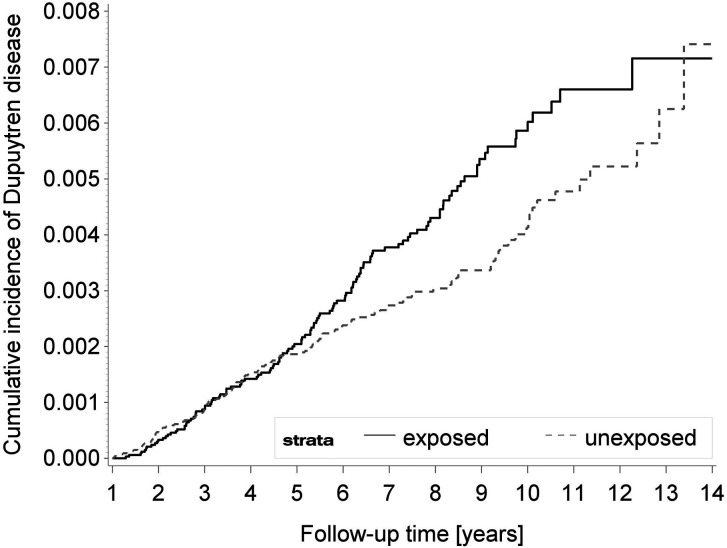
Cumulative incidences of Dupuytren’s disease (DD) in exposed patients to bariatric surgery and unexposed patients.

Cumulative incidence of DD in exposed and unexposed patients over time is shown in Figure 3. We observed an increased incidence of DD in exposed patients especially seen beyond 5 years of follow-up.

## Discussion

In this large population-based, retrospective cohort study of 34,959 patients undergoing bariatric surgery in Sweden, we observed a 30% increased risk of incident DD following surgery to reduce BMI when matched to 54,769 unexposed episodes. A maximum follow-up time of 13 years within the study also enabled identification of an increased risk of DD as the difference in incidence increased over the long follow-up period available.

In our study, we showed that weight loss induced by bariatric surgery was associated with an increased incidence of DD, particularly after allowing time for weight loss to exert its metabolic effects. These results are consistent with previous studies that have shown obesity to be causally protective against DD (Majeed et al., 2021).

Two sensitivity analyses merit further discussion. First, we observed that the risk of DD was higher in women rather than men. We think that this slightly counter-intuitive result (as DD is well-known to be more common in men than women) is caused by two factors. First, 72% of our exposed cohort was female (Table 1). Second, the observation time in females was 173.9 × 1,000 person-years, but for males it was 66.1 × 1000 person-years (Table 2). The effect of bariatric surgery on development of DD also takes several years to manifest and continues to increase over time (Figure 3). These factors combined also meant there was more statistical power to observe an effect in females compared with males. Second, the risk of DD did not differ between age groups. As with sex, this discrepancy may be caused by the increased number of younger people in the study. We note that after propensity score-matching, the mean age of the exposed and unexposed cohorts was 45.5 and 45.9 years, respectively (Table 1). Similarly, the observation time in the 30–54 age group was 194.4 × 1000 person-years, but for the older age group it was 42.5 × 1000 person-years (Table 2). These factors meant that while there were more absolute events in the younger age group, the risk of developing DD was similar between these two groups in the sensitivity analysis (HR 1.38 and 1.37, respectively; Table 2).

The reasons for a higher incidence of DD with decreasing obesity remains unclear. One potential mechanism, as previously discussed (Majeed et al., 2021), may be that adipose tissues in the palm suppress pathological myofibroblast activity, which may also account for the relative success of percutaneous aponeurotomy with lipofilling (PALF), a minimally invasive technique that in a randomized controlled trial showed equivalent outcomes to limited fasciectomy at 1-year follow-up (Kan et al., 2016). In addition, adipose-derived stem cells have also been shown to suppress fibroproliferation and reduce contraction of DD-derived myofibroblasts (Verhoekx et al., 2013).

This study used nationwide data with known high data quality and established external validity, which ensured a large study sample with long and complete follow-up. Use of linked data within the registries also enabled a fuller picture of patient demographics to be generated and enabled a propensity score-matched study design. This led to the creation of well-balanced groups, and also balanced any censoring criteria. Moreover, the use of sensitivity analyses with increased control for confounding suggested that robust results were yielded. Finally, a matched unexposed patient started follow-up at the random cohort entry date within the same entry block as their matched exposed patient. Since this happened within the same entry block as the matched exposed patient (i.e. a maximum of 4 years apart of one another), bias was prevented through time trends in health care delivery, for example, through increasing frequency of bariatric surgery over time. This entry block design therefore helped to maximize comparability between matched patients.

Despite the rigorous methodology of this study, our results must be interpreted in the context of the following limitations. While patients with obesity unexposed to surgery were identified using the relevant ICD code, actual BMI measurements in unexposed patients were not available. Thus, there could be a potential baseline difference in BMI between the exposed and unexposed groups. However, we observed a higher prevalence of risk factors associated with obesity (e.g. type 2 diabetes, hypertension, cardiovascular disease) in the unexposed group before propensity score-matching, and all these risk factors were balanced after propensity score-matching. Thus, it is a credible assumption that BMI was also sufficiently balanced along with obesity-related conditions.

This study assessed the risk of DD following bariatric surgery in a large population-based registry. Our findings are consistent with previous epidemiological and genetic data demonstrating that obesity is protective in the patho-aetiology of DD (Hacquebord et al., 2017; Majeed et al., 2021; Major et al., 2019), a most unusual biological phenomenon. The latency of risk increase of DD after bariatric surgery may suggest that slowly adapting metabolic changes may be part of the mechanism of DD emergence. Future research should focus upon the interplay of DD with metabolic syndrome, in order to identify potential biological mechanisms and therapeutic targets.

## Supplemental Material

sj-pdf-1-jhs-10.1177_17531934211062023 - Supplemental material for The association of bariatric surgery and Dupuytren’s disease: a propensity score-matched cohort study Click here for additional data file. Supplemental material, sj-pdf-1-jhs-10.1177_17531934211062023 for The association of bariatric surgery and Dupuytren’s disease: a propensity score-matched cohort study by Theresa Burkard, Jennifer C. E. Lane, Dag Holmberg, Anders Thorell, Andrea M Burden and Dominic Furniss in Journal of Hand Surgery (European Volume)

sj-pdf-2-jhs-10.1177_17531934211062023 - Supplemental material for The association of bariatric surgery and Dupuytren’s disease: a propensity score-matched cohort study Click here for additional data file. Supplemental material, sj-pdf-2-jhs-10.1177_17531934211062023 for The association of bariatric surgery and Dupuytren’s disease: a propensity score-matched cohort study by Theresa Burkard, Jennifer C. E. Lane, Dag Holmberg, Anders Thorell, Andrea M Burden and Dominic Furniss in Journal of Hand Surgery (European Volume)

sj-pdf-3-jhs-10.1177_17531934211062023 - Supplemental material for The association of bariatric surgery and Dupuytren’s disease: a propensity score-matched cohort study Click here for additional data file. Supplemental material, sj-pdf-3-jhs-10.1177_17531934211062023 for The association of bariatric surgery and Dupuytren’s disease: a propensity score-matched cohort study by Theresa Burkard, Jennifer C. E. Lane, Dag Holmberg, Anders Thorell, Andrea M Burden and Dominic Furniss in Journal of Hand Surgery (European Volume)

## References

[bibr1-17531934211062023] Alser OH Kuo RYL Furniss D (2020) Nongenetic factors associated with Dupuytren's disease: a systematic review. Plast Reconstr Surg 146: 799–807. 32970002 10.1097/PRS.0000000000007146

[bibr2-17531934211062023] Brooke HL Talbäck M Hörnblad J , et al.(2017) The Swedish cause of death register. Eur J Epidemiol 32: 765–73. 28983736 10.1007/s10654-017-0316-1 PMC5662659

[bibr3-17531934211062023] Buchwald H Avidor Y Braunwald E , et al.(2004) Bariatric surgery: a systematic review and meta-analysis. JAMA 292: 1724–37. 15479938 10.1001/jama.292.14.1724

[bibr4-17531934211062023] Eaton CB Hartman SJ Perzanowski E , et al.(2016) A randomized clinical trial of a tailored lifestyle intervention for obese, sedentary, primary care patients. Ann Fam Med 14: 311–9. 27401418 10.1370/afm.1952 PMC4940460

[bibr5-17531934211062023] Franklin JM Rassen JA Ackermann D Bartels DB Schneeweiss S (2014) Metrics for covariate balance in cohort studies of causal effects. Stat Med 33: 1685–99. 24323618 10.1002/sim.6058

[bibr6-17531934211062023] Geoghegan JM Forbes J Clark DI Smith C Hubbard R (2004) Dupuytren's disease risk factors. J Hand Surg Br 29: 423–6. 15336742 10.1016/j.jhsb.2004.06.006

[bibr7-17531934211062023] Gill H Kang S Lee Y , et al.(2019) The long-term effect of bariatric surgery on depression and anxiety. J Affect Disord 246: 886–94. 30795495 10.1016/j.jad.2018.12.113

[bibr8-17531934211062023] Gudmundsson KG Arngrímsson R Sigfússon N Björnsson A Jónsson T (2000) Epidemiology of Dupuytren's disease: clinical, serological, and social assessment. The Reykjavik study. J Clin Epidemiol 53: 291–6. 10760640 10.1016/s0895-4356(99)00145-6

[bibr9-17531934211062023] Hacquebord JH Chiu VY Harness NG (2017) The risk of Dupuytren diagnosis in obese individuals. J Hand Surg Am 42: 149–55. 28111059 10.1016/j.jhsa.2016.12.010

[bibr10-17531934211062023] Hart MG Hooper G (2005) Clinical associations of Dupuytren's disease. Postgrad Med J 81: 425–8. 15998816 10.1136/pgmj.2004.027425 PMC1743313

[bibr11-17531934211062023] Hernán MA Hernández-Díaz S Werler MM Mitchell AA (2002) Causal knowledge as a prerequisite for confounding evaluation: an application to birth defects epidemiology. Am J Epidemiol 155: 176–84. 11790682 10.1093/aje/155.2.176

[bibr500-17531934211062023] Kan HJ, Selles RW, van Nieuwenhoven CA, Zhou C, Khouri RK, Hovius SER. Percutaneous Aponeurotomy and Lipofilling (PALF) versus Limited Fasciectomy in Patients with Primary Dupuytren's Contracture: A Prospective, Randomized, Controlled Trial. Plast Reconstr Surg. 2016, 137: 1800–12. 10.1097/PRS.0000000000002224 27219235

[bibr12-17531934211062023] Kuo RYL Ng M Prieto-Alhambra D Furniss D (2020) Dupuytren's disease predicts increased all-cause and cancer-specific mortality: analysis of a large cohort from the U.K. clinical practice research datalink. Plast Reconstr Surg 145: 574e–82e. 10.1097/PRS.0000000000006551 PMC7043723 32097318

[bibr13-17531934211062023] Ludvigsson JF Andersson E Ekbom A , et al.(2011) External review and validation of the Swedish National Inpatient Register. BMC Public Health 11: 450. 21658213 10.1186/1471-2458-11-450 PMC3142234

[bibr14-17531934211062023] Ludvigsson JF Otterblad-Olausson P Pettersson BU Ekbom A (2009) The Swedish personal identity number: possibilities and pitfalls in healthcare and medical research. Eur J Epidemiol 24: 659–67. 19504049 10.1007/s10654-009-9350-y PMC2773709

[bibr15-17531934211062023] Majeed M Wiberg A Ng M Holmes MV Furniss D (2021) The relationship between body mass index and the risk of development of Dupuytren's disease: a Mendelian randomization study. J Hand Surg Eur 46: 406–10. 10.1177/1753193420958553 32972297

[bibr16-17531934211062023] Major M Freund MK Burch KS , et al.(2019) Integrative analysis of Dupuytren's disease identifies novel risk locus and reveals a shared genetic etiology with BMI. Genet Epidemiol 43: 629–45. 31087417 10.1002/gepi.22209 PMC6699495

[bibr17-17531934211062023] Nordic Co-operation. Nomesco classification of surgical procedures. 2021. http://norden.diva-portal.org/smash/record.jsf?pid=diva2%3A968721&dswid=1487 (accessed 12 March 2021).

[bibr18-17531934211062023] Parsons L (2001) Reducing bias in a propensity score matched-pair sample using greedy matching techniques. Proc Twenty-Sixth Annual SAS Users 214–26.

[bibr19-17531934211062023] Schauer DP Feigelson HS Koebnick C , et al.(2019) Bariatric surgery and the risk of cancer in a large multisite cohort. Ann Surg 269: 95–101. 28938270 10.1097/SLA.0000000000002525 PMC6201282

[bibr20-17531934211062023] SOReg. Scandinavian obesity surgery registry. 2021. https://www.ucr.uu.se/soreg/ (accessed 22 February 2021).

[bibr21-17531934211062023] Tao W Holmberg D Näslund E , et al.(2016) Validation of obesity surgery data in the Swedish National Patient Registry and Scandinavian Obesity Registry (SOReg). Obes Surg 26: 1750–6. 26667162 10.1007/s11695-015-1994-y

[bibr80-17531934211062023] Verhoekx JSN, Mudera V, Walbeehm ET, Hovius SER. Adipose-derived stem cells inhibit the contractile myofibroblast in Dupuytren's disease. Plast Reconstr Surg. 2013, 132: 1139–48. 10.1097/PRS.0b013e3182a3bf2b 23924646

[bibr22-17531934211062023] WHO. International classification of disease version 10. 2016. https://www.who.int/classifications/icd/icdonlineversions/en/ (accessed 15 October 2018).

[bibr23-17531934211062023] WHO. Obesity and overweight. WHO, 2020. https://www.who.int/news-room/fact-sheets/detail/obesity-and-overweight (accessed 22 February 2021).

