# Supplementary material for: The association of bariatric surgery and Dupuytren’s disease: a propensity score-matched cohort study
Source: J Hand Surg Eur Vol. 2021 Dec 1;47(3):288–95. doi: 10.1177/17531934211062023 (PMC8892052; doi:10.1177/17531934211062023)
Supplement: sj-pdf-1-jhs-10.1177_17531934211062023 - Supplemental material for The association of bariatric surgery and Dupuytren’s disease: a propensity score-matched cohort study [file sj-pdf-1-jhs-10.1177_17531934211062023.pdf]

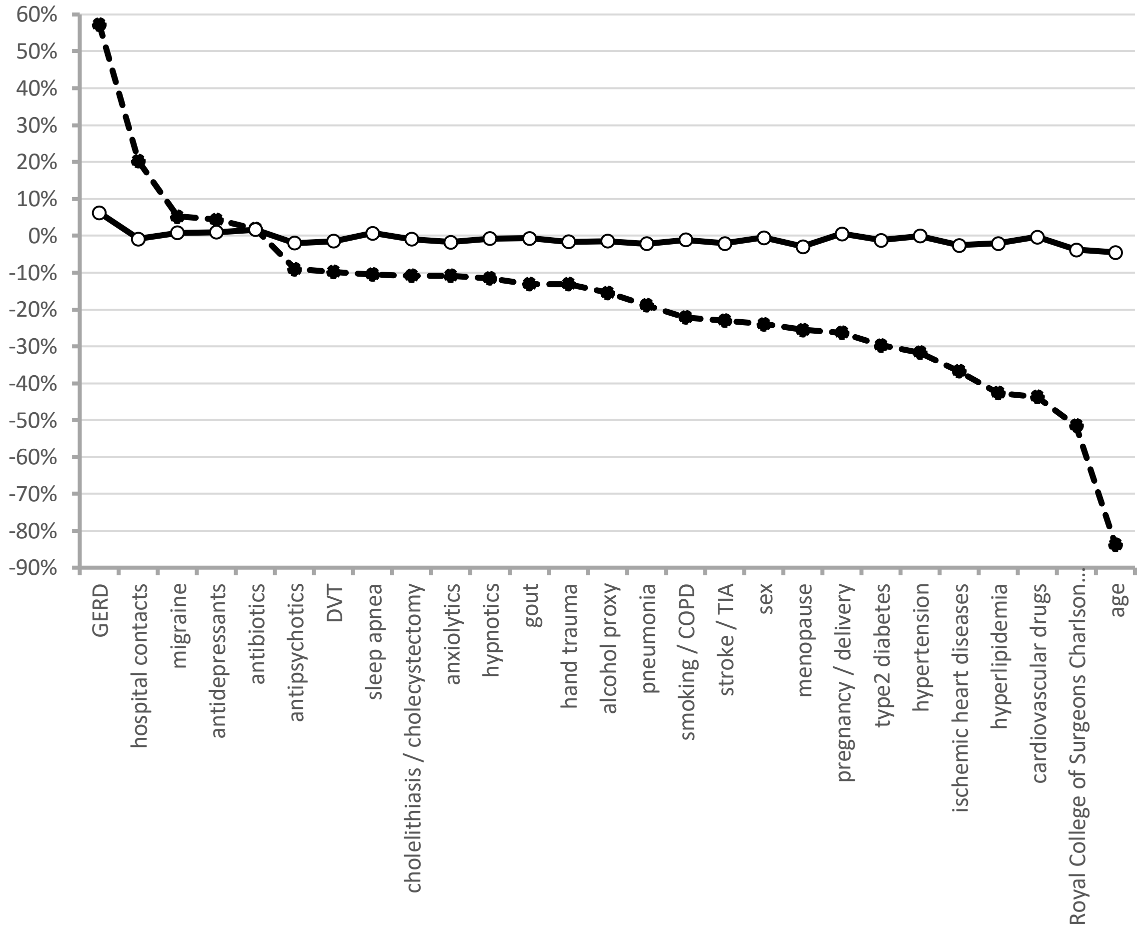

GERD= gastroesophageal reflux disease; DVT = deep vein thrombosis; Royal College of Surgeons Charlson= Royal College of Surgeons Charlson Comorbidity Index

Supplementary Figure 1. Standardized mean differences of covariates before (filled dots) and after (open dots) Propensity Score (PS)-matching
