# Supplementary material for: The association of bariatric surgery and Dupuytren’s disease: a propensity score-matched cohort study
Source: J Hand Surg Eur Vol. 2021 Dec 1;47(3):288–95. doi: 10.1177/17531934211062023 (PMC8892052; doi:10.1177/17531934211062023)
Supplement: sj-pdf-2-jhs-10.1177_17531934211062023 - Supplemental material for The association of bariatric surgery and Dupuytren’s disease: a propensity score-matched cohort study [file sj-pdf-2-jhs-10.1177_17531934211062023.pdf]

Supplementary Table 1. Body mass index at bariatric surgery and 1.2. and 5 years thereafter overall and stratified by surgery type

Overall	Overall exposed (N=43 780) before PS-matching	Overall exposed (N=34 959) after PS-matching	Exposed within subgroups of surgery (re-matched)
BMI at surgery [kg/m^2] (SD) [a]	42.1 (5.5)	42.1 (5.6)	NA
BMI at 1 year after surgery(SD) [b]	29.1 (4.7)	29.3 (4.7)	NA
BMI at 2 year after surgery (SD) [c]	29.0 (4.9)	29.1 (4.9)	NA
BMI at 5 year after surgery (SD) [d]	30.4 (5.2)	30.6 (5.2)	NA
Sleeve gastrectomy	6065 (13.8%)	4470 (12.8%)	6065
BMI at surgery (SD)	40.9 (5.6)	40.9 (5.8)	40.9 (5.6)
BMI at 1 year after surgery (SD)	30.3 (5.1)	30.5 (5.3)	30.3 (5.1)
BMI at 2 year after surgery (SD)	30.7 (5.3)	30.9 (5.4)	30.7 (5.3)
BMI at 5 year after surgery (SD)	33.0 (6.4)	32.9 (6.2)	33.0 (6.4)
Gastric bypass	37409 (85.3%)	30156 (86.3%)	37409
BMI at surgery (SD)	42.1 (5.2)	42.1 (5.3)	42.1 (5.2)
BMI at 1 year after surgery (SD)	28.9 (4.6)	29.1 (4.6)	28.9 (4.6)
BMI at 2 year after surgery (SD)	28.7 (4.7)	28.9 (4.7)	28.7 (4.7)
BMI at 5 year after surgery (SD)	30.3 (5.1)	30.4 (5.1)	30.3 (5.1)
Duodenal switch	396 (0.9%)	333 (1.0%)	396
BMI at surgery (SD)	55.1 (7.5)	55.2 (7.2)	55.1 (7.5)

BMI at 1 year after surgery (SD)	33.4 (6.1)	33.4 (6.0)	33.4 (6.1)
BMI at 2 year after surgery (SD)	31.6 (5.9)	31.7 (6.1)	31.6 (5.9)
BMI at 5 year after surgery (SD)	33.0 (6.1)	33.0 (6.2)	33.0 (6.1)

BMI: body mass index [kg/m^2]; SD: standard deviation.

[a] 5.6% of surgeries have missing BMI data

[b] 14.5% of surgeries have missing BMI data

[c] 30.1% of surgeries have missing BMI data

[d] 58.9% of surgeries have missing BMI data

Supplementary Table 2. Censoring reasons before and after PS-matching

	Before PS-matching		PS-matched	
	**Exposed (N=43 780)**	**Unexposed (N=317 205)**	**Exposed (N=34 959)**	**Unexposed (N=54 769)**
End of study period	42471 (96.8%)	265331 (83.7%)	33700 (96.4%)	47950 (87.6%)
Death	1149 (2.6%)	36774 (11.6%)	1045 (3%)	2320 (4.2%)
Loss to follow-up	108 (0.3%)	459 (0.1%)	86 (0.3%)	94 (0.2%)
Change in exposure status	3 (0%)	13684 (4.3%)	2 (0%)	4269 (7.8%)
Outcome (Dupuytren's Disease)	139 (0.3%)	957 (0.3%)	126 (0.4%)	136 (0.3%)

Supplementary Table 3. Results of the association of DD after bariatric surgery and DD overall and in subgroups

	Before PS-matching (cox regression analysis)				PS-matched		
	**Obs.-time[a] in bar. surg. pat. + unexposed**	**Events in bar. surg. pat. + unexposed**	**HR crude (95% CI)**	**HR adjusted[b] (95% CI)**	**Obs.-time[a] in bar. surg. pat. + unexposed**	**Events in bar. surg. pat. + unexposed**	**HR matched[b] (95% CI)**
Overall	298.8+1882.6	139+957	0.89 (0.74-1.06)	1.24 (1.02-1.51)	241.8+345.3	126+136	1.30 (1.02-1.65)
Sex							
**Women**	222.6+1190.3	93+414	1.16 (0.92-1.45)	1.33 (1.03-1.73)	173.9+242.8	83+83	1.36 (1.00-1.84)
**Men**	76.2+692.2	46+543	0.75 (0.56-1.02)	1.10 (0.79-1.52)	66.1+99.5	40+56	1.05 (0.70-1.58)
Age in years							
**30-54**	254.3+948.0	94+256	1.31 (1.04-1.66)	1.24 (0.94-1.62)	194.4+267.8	79+77	1.38 (1.01-1.88)
**55-79**	44.5+41.0	45+701	1.32 (0.98-1.79)	1.23 (0.89-1.69)	42.5+8.6	42+52	1.37 (0.92-2.06)
Bariatric surgery type							
**Restrictive surgery**	23.6+1882.6	8+957	0.76 (0.38-1.52)	1.08 (0.54-2.19)	21.5+38.7	8+15	1.01 (0.43-2.38)
**Malabsorptive surgery**	272.6+1882.6	130+957	0.90 (0.75-1.08)	1.26 (1.02-1.54)	225.6+324.8	121+128	1.33 (1.04-1.71)
**Combined restrictive and malabsorptive surgery**	2.6+1882.6	1+957	NA	NA	2.6+4.7	1+2	NA
Duration of follow-up							
**>1-5 years**	193.3+1290.8	67+570	0.77 (0.59-0.99)	1.11 (0.84-1.47)	155.0+230.3	60+82	1.07 (0.76-1.49)
**>5-13 years**	105.4+591.8	72+387	1.05 (0.81-1.34)	1.40 (1.05-1.87)	76.8+101.4	66+54	1.63 (1.14-2.34)
Sensitivity analyses							
**1-5 years**	194.8+1314.1	78+786	0.67 (0.53-0.84)	1.07 (0.83-1.38)	158.9+227.9	65+101	0.92 (0.67-1.25)
**2-year run-in period**	293.9+192.3	123+792	0.92 (0.76-1.12)	1.28 (1.03-1.59)	236+342.4	107+108	1.40 (1.07-1.83)
**>2-5 years**	188.5+1229.9	51+405	0.80 (0.60-1.07)	1.14 (0.83-1.58)	149.9+225.5	44+54	1.20 (0.80-1.78)
**Trimmed PS**	NA	NA	NA	NA	127.5+220.1	69+78	1.49 (1.08-2.07)
