# Supplementary material for: The association of bariatric surgery and Dupuytren’s disease: a propensity score-matched cohort study
Source: J Hand Surg Eur Vol. 2021 Dec 1;47(3):288–95. doi: 10.1177/17531934211062023 (PMC8892052; doi:10.1177/17531934211062023)
Supplement: sj-pdf-3-jhs-10.1177_17531934211062023 - Supplemental material for The association of bariatric surgery and Dupuytren’s disease: a propensity score-matched cohort study [file sj-pdf-3-jhs-10.1177_17531934211062023.pdf]

Supplementary Methods 1: Description of covariates used for PS-matching

Covariates used for matching included medical diagnoses recorded at any time before cohort entry and dispensation of pharmacologic agents was recorded within 6 months before cohort entry. Selected diagnoses and medications were associated with BMI (e.g. type 2 diabetes, hypertension, ischemic heart disease), with undergoing surgery in general (e.g. Royal College of Surgeons Charlson Comorbidity Score, sleep apnoea), with the risk of DD (e.g. hand trauma, alcohol consumption, smoking), or were potential confounders of the association between bariatric surgery and DD (e.g. age, sex) (Alser et al., 2020; Armitage and van der Meulen, 2010). Covariates further included proxies for patient frailty (e.g. pneumonia) or engagement with the health care system (number of hospital encounters ≤1 year prior to cohort entry).

All covariates used for matching are detailed in Table 1 in the main body.

References:

Alser OH, Kuo RYL, Furniss D. Nongenetic factors associated with dupuytren's disease: A systematic review. Plast Reconstr Surg. 2020, 146: 799-807.

Armitage JN, van der Meulen JH. Identifying co-morbidity in surgical patients using administrative data with the royal college of surgeons charlson score. Br J Surg. 2010, 97: 772-81.
